# Improving Signal-Strength Aggregation for Mobile Crowdsourcing Scenarios

**DOI:** 10.3390/s21041084

**Published:** 2021-02-05

**Authors:** Diego Madariaga, Javier Madariaga, Javier Bustos-Jiménez, Benjamin Bustos

**Affiliations:** 1 NIC Chile Research Labs, University of Chile, Santiago 8320000, Chile; javier@niclabs.cl (J.M.); jbustos@niclabs.cl (J.B.-J.); 2 Department of Computer Science, Millennium Institute Foundational Research on Data, University of Chile, Santiago 8320000, Chile; bebustos@dcc.uchile.cl

**Keywords:** signal strength, mobile crowdsourcing, network measurements

## Abstract

Due to its huge impact on the overall quality of service (QoS) of wireless networks, both academic and industrial research have actively focused on analyzing the received signal strength in areas of particular interest. In this paper, we propose the improvement of signal-strength aggregation with a special focus on Mobile Crowdsourcing scenarios by avoiding common issues related to the mishandling of log-scaled signal values, and by the proposal of a novel aggregation method based on interpolation. Our paper presents two clear contributions. First, we discuss the misuse of log-scaled signal-strength values, which is a persistent problem within the mobile computing community. We present the physical and mathematical formalities on how signal-strength values must be handled in a scientific environment. Second, we present a solution to the difficulties of aggregating signal strength in Mobile Crowdsourcing scenarios, as a low number of measurements and nonuniformity in spatial distribution. Our proposed method obtained consistently lower Root Mean Squared Error (RMSE) values than other commonly used methods at estimating the expected value of signal strength over an area. Both contributions of this paper are important for several recent pieces of research that characterize signal strength for an area of interest.

## 1. Introduction

During the last decade, many research studies have made use of Mobile Crowdsourcing methods to analyze the performance and quality of service (QoS) in mobile environments. These studies usually obtain different QoS indicators together with some environmental data such as timestamps, location coordinates and cell identifiers, to describe wireless network behavior for a given geographical area. Among all the collected network information, the received signal-strength indicator is included in most Mobile Crowdsourcing analyses. This recurrent consideration of signal strength is in part because it is very easy to obtain from end-user mobile devices [1], but mostly because of its influence on the overall QoS in wireless networks, which is reflected on the impact produced by signal-strength variations in network performance measurements [1,2,3,4]. Moreover, analyses over signal-strength data are not only interesting for academic research, but also for mobile analytics companies as *OpenSignal* and *Tutela*, and for mobile network operators for radio network planning [5,6] and for performing coverage analysis in cellular networks [7].

A common methodology to summarize the received signal strength inside a specific area is to aggregate all the individual measurements into one representative value that characterizes the signal strength inside the location area [1,8,9,10,11,12,13,14,15,16,17,18].

The first question about getting a representative signal-strength value from the aggregation of several individual measurements is to identify what real value we actually want to represent and estimate. In this paper, we consider the formal definition of the expected value of signal strength as the target value to be estimated. The expected value is a measure of central tendency, i.e., a value for which the results will tend to. Intuitively, it is the theoretical mean value of a random variable over a large number of experiments, and it is commonly used to summarize all the information about a random variable in a single numerical value.

In Mobile Crowdsourcing scenarios, signal-strength samples are taken by real end-user devices with custom measurement apps. This leads to important sources of error to take into account when aggregating values inside an area:

1. Measurements are not uniformly distributed in the area, as they are defined by human mobility patterns [19].
2. The number of measurements in small areas (e.g., coverage area of a single cell) could not be high enough to be considered representative enough [1].
3. The measurements present accuracy errors in both signal-strength values and geographic coordinates [1].

Hence, some commonly used methods to characterize signal strength could not necessarily return a good estimation of expected value of signal strength, since they do not take into consideration the sources of error aforementioned, which are present in most Mobile Crowdsourcing signal-strength data.

Our paper presents two clear contributions. First, we present a formal analysis about how signal-strength values must be handled to avoid some common pitfalls in using log-scaled signal-strength. Second, we present a novel aggregation method based on interpolation of signal strength (ABOI method). Our proposed method obtained consistently lower RMSE values than other commonly used methods at estimating the expected value of signal strength over an area, in both simulated and real scenarios. Consequently, the ABOI method is demonstrated to be more robust against the existing difficulties of real-world measurements.

The rest of this paper is structured as follows. In Section 2, we discuss the literature on aggregating signal strength for a variety of different purposes. Section 3 presents the physical and mathematical formalities regarding how signal-strength values must be handled when applying mathematical operations. In Section 4, we discuss the most used methods to aggregate signal-strength measurements in Mobile Crowdsourcing scenarios, and their unsuitability when dealing with low number of measurements and nonuniformity in spatial distribution. We also present a novel aggregation method based on interpolation of signal strength, to face these real-world difficulties. Section 5 provides the mathematical foundation for using our proposed method to estimate the expected value of signal strength inside an area of interest. Section 6 indicates that our proposed method obtains better results than other commonly used methods for aggregating signal strength, in both simulated and real scenarios. We conclude in Section 7 that for most Mobile Crowdsourcing scenarios, our proposed model based on interpolation should be preferred over the other methodologies, since it has a better performance even when the number of measurements is low and the spatial distribution of the samples is nonuniform, which is a typical case for real Mobile Crowdsourcing data.

## 2. Related Work

Due to its large impact on the overall QoS of wireless networks, many research works have focused on characterizing the received signal strength for an area of particular interest. These analyses frequently used all the individual signal-strength samples taken by each mobile device sensing the network.

In this way, some works aggregated several signal-strength samples inside the same area into a unique representative signal-strength value to predict user availability [9], measure the effect of weather conditions in the received signal strength [10,11], analyze network performance [12], measure the impact on signal strength of indoor-outdoor context [13] and find correlations between signal strength and other QoS indicators for mobile networks as network congestion [15], throughput [8] and TCP goodput and latency inside the same geographic area [1]. In this paper, we propose an aggregation method that better estimates the expected value of signal strength than the methods used in the papers mentioned above, especially when using measurements taken in Mobile Crowdsourcing contexts. Thus, the results and conclusions of these works can be refined by using our proposed model.

It is important to notice that there are other research works that also aggregated several signal-strength samples, although not to find a representative signal-strength value. Some of these works used signal aggregation to perform base transceiver station (BTS) localization [20] or to estimate user location [14,21], mostly based on the RADAR system [22]. These studies are not that related to the problem we are referring to in this paper, which is to aggregate signal strength into a representative value to estimate the mathematical expectation of the signal strength.

There are some works that developed high-resolution coverage maps from Mobile Crowdsourcing signal-strength measurements. These maps were created by plotting each empirical sample on the map [23,24] or by interpolating the signal strength in several uniformly distributed points inside the area of interest using linear interpolation [25], using variations of Kriging method [26,27] or by using Gaussian processes that consider a prior knowledge about theoretical path loss models [28]. These coverage maps are useful for tasks that require highly detailed maps, but when analyzing signal coverage in greater areas, the effectiveness of their fine-grained visualizations will decrease as the resolution of the maps decreases. Positioning all individual samples will greatly increase the clutter in the visualizations, defeating the purpose of providing useful information for the measured areas. Therefore, for these cases, it is also important to take into account the aggregation of signal-strength samples, to be able to generalize their results to maps with lower resolution, where it will be convenient to represent the signal strength in areas by only one representative value. Consequently, the method for signal-strength aggregation proposed in this paper could also be useful for these works.

Some works that employed Mobile Crowdsourcing data discussed the problem of not having uniformly distributed samples in the measured area [26,28] and how the spatial distribution of the samples matches population patterns [29]. This is important, since some researchers that used simulated Mobile Crowdsourcing data to evaluate their methods, implicitly assumed uniform spatial distribution of the samples (as shown in Section 4.1). Uniform distribution is not a realistic measurement scenario, especially when samples are taken by real end-user mobile devices. For a better reliability of this paper’s results, we consider in our experiments both uniform distribution of signal-strength samples and distribution based on social network theory [19], which is closer to the spatial distribution present in real Mobile Crowdsourcing measurements.

## 3. Common Pitfalls in Using Log-Scaled Signal Strength

The use of log-scaled signal-strength values is a widespread methodology for analyzing radio frequency measurements. Signal amplitude could vary very widely, and therefore, it could be difficult to analyze and understand the relationships among different values in the linear watt scale. Hence, using log-scale enhances signal-strength visualizations by improving the display range of axes. The use of log-scaled signal-strength values is also attractive since it can lead to a compression of data, requiring fewer bits of information [30].

Log-scaled signal-strength values could be used in dBm units (decibels with reference to one milliwatt) or in Arbitrary Strength Units (ASU), since ASU values are linearly proportional to the received signal strength in dBm, and consequently, they are also logarithmic values.

Before using and manipulating dBm values, it is important to analyze the origin of dBm from a physical point of view, regarding *dimensional analysis*. Power is a derived quantity that can be expressed in terms of fundamental units (time, length, and mass). In fact, power values must have dimension $ML^{2}T^{-3}$. The International System of Units (SI) describes the watt (symbol: W) as a unit of power, defined as a derived unit in terms of base units, where $1\;W=1\;\mathrm{kg}\cdot m^{2}\cdot s^{-3}$. In addition, the prefix *Milli-* (symbol m) has been part of the SI since 1960, and it only denotes a factor of $10^{-3}$. This prefix never changes the units, and therefore, values expressed in milliwatt (mW) are actually being expressed in watt (W) units.

If we consider a power value $P_{mW}$ expressed in milliwatts, its corresponding value in dBm is formulated as

(1) $$P_{dBm}=10\log_{10}\left(\frac{P_{mW}}{1\;\mathrm{mW}}\right)$$

It is clear to see that the right side in Equation (1) is dimensionless, since mW dimension is canceled in $\frac{P_{mW}}{1\;\mathrm{mW}}$. Then, the parameter inside the logarithm function in Equation (1) is a dimensionless number, and therefore, $P_{dBm}$ is also dimensionless. This fact is essential because, even when $P_{mW}$ has dimensions, there is no physical sense for $P_{dBm}$ to have it. Thus, it is a mistake to consider dBm as a power unit, since it does not meet the dimension of power quantities ($ML^{2}T^{-3}$).

The above is documented by Sonin [31]: “*Products, ratios, powers, and exponential and other functions such as trigonometric functions and **logarithms** are defined for numbers, but **have no physical correspondence in operations involving actual physical quantities***”.

Furthermore, the mere fact that $P_{dBm}$ is defined as a logarithmic function implies that dBm is a dimensionless quantity. In fact, we consider the formal definition of $10\log_{10}\left(x\right)$:

$$10\log_{10}\left(x\right)=\frac{10}{\ln(10)}\int_{t=1}^{t=x}\frac{dt}{t}$$

The integral $\int_{t=1}^{t=x}\frac{dt}{t}$ corresponds to the sum of an infinite number of terms $\frac{dt}{t}$. All these terms are dimensionless, and therefore, the whole expression $10\log_{10}\left(x\right)$ will always be dimensionless. Then, further interpretations of the dimensionality of dBm are not accepted: dBm values are intrinsically dimensionless.

This non-coherency in dimensionality between dBm values and power quantities ($ML^{2}T^{-3}$) can be demonstrated with the knowledge of the use of dimensional formulas in changing units [32], where there is no possible transformation to consistently change from W to dBm. The consequence of the aforementioned is that dBm values do not meet *Bridgman’s principle of absolute significance of relative magnitude* (Lemma 1), which is essential to all the systems of measurement in scientific use [33].

**Lemma** **1.** *dBm values **do not** meet Bridgman’s principle of absolute significance of relative magnitude.*

**Proof.**  Let SQ be a secondary quantity described by

SQ=f(α,β,γ,…),

where α,β,γ,… are primary quantities and *f* is the function that combines them.

SQ satisfies Bridgman’s principle of absolute significance of relative magnitude if

(2) $$\frac{f(\alpha_{1},\beta_{1},\gamma_{1},\ldots )}{f(\alpha_{2},\beta_{2},\gamma_{2},\ldots )}=\frac{f(x\alpha_{1},y\beta_{1},z\gamma_{1},\ldots )}{f(x\alpha_{2},y\beta_{2},z\gamma_{2},\ldots )}$$

holds for all values of $\alpha_{1},\;\beta_{1},\;\gamma_{1},\ldots ,\;\alpha_{2},\;\beta_{2},\;\gamma_{2},\ldots$ and for all coefficients x,y,z,… [33].

As stated in Equation (1), dBm can be described as a function of primary quantities:

(3) $$f_{dBm}\left(\alpha ,\beta ,\gamma \right)=10\log_{10}\left(\frac{\alpha \left[kg\right]\cdot \beta \left[m^{2}\right]\cdot \gamma \left[s^{-3}\right]}{10^{-3}\cdot 1\left[kg\right]\cdot 1\left[m^{2}\right]\cdot 1\left[s^{-3}\right]}\right)$$

Proceeding by contradiction, assume that dBm values do meet Bridgman’s principle of absolute significance of relative magnitude. Then, Equation (2) should hold for $f_{dBm}$ in Equation (3) and for all values of $\left(\alpha_{1},\beta_{1},\gamma_{1}\right)$, $\left(\alpha_{2},\beta_{2},\gamma_{2}\right)$ and (x,y,z). In particular, it should hold for the following values:

$$\begin{array}{lll} \alpha_{1}=10^{-9}\;\;\;\;\;\; & \beta_{1}=1\;\;\;\;\;\; & \gamma_{1}=1\;\;\;\;\;\;\\ \alpha_{2}=10^{-8}\;\;\;\;\;\; & \beta_{2}=1\;\;\;\;\;\; & \gamma_{2}=1\;\;\;\;\;\;\\ x=10\;\;\;\;\;\; & y=1\;\;\;\;\;\; & z=1\;\;\;\;\;\; \end{array}$$

By replacing these values in the left side of Equation (2):

$$\frac{f_{dBm}\left(\alpha_{1},\beta_{1},\gamma_{1}\right)}{f_{dBm}\left(\alpha_{2},\beta_{2},\gamma_{2}\right)}=\frac{-60}{-50}=1.2$$

And by replacing these values in the right side of Equation (2):

$$\frac{f_{dBm}\left(x\alpha_{1},y\beta_{1},z\gamma_{1}\right)}{f_{dBm}\left(x\alpha_{2},y\beta_{2},z\gamma_{2}\right)}=\frac{-50}{-40}=1.25$$

Both sides of the equation are not equal, which is a contradiction. Then, since the relationship is not fulfilled for all values, we conclude that dBm values (represented as $f_{dBm}$) do not satisfy Bridgman’s principle of absolute significance of relative magnitude. □

As a direct consequence of Lemma 1, equations involving dBm units are considered to be not physically relevant [31].

Meeting Bridgman’s principle is, according to Percy Bridgman [33], essential to all the systems of measurement in scientific use. This principle is fundamental to guarantee that the selection of a different unit of measurement will not affect the outcomes of any experiment. Therefore, as dBm values do not meet Bridgman’s principle, some numerical relationships among power values in W do not remain true when using dBm, i.e., the outcomes of scientific experiments can be affected if using dBm values instead of watt values. This should not be allowed in scientific research, as nature is indifferent to the arbitrary choices we make when we pick base units. Indeed, as Sonin [31] precisely stated: “*Nature is indifferent to the arbitrary choices we make when we pick base units. We are interested, therefore, only in numerical relationships that remain true independent of base unit size.*”. However, dBm values do not respect this, as demonstrated in the following straightforward example:

1mW+1mW=2mW

If we transform all values from mW to dBm using Equation (1), we have

0dBm+0dBm=3.0102999566dBm

This is, of course, wrong and contradictory, and it exemplifies that if we wrongly attempt to perform addition of dBm values, we will reach erroneous conclusions such as 1mW=2mW. Accordingly, to be coherent with the dimensional analysis and with the mathematical basis, all mathematical operations involving signal strength must use linear watt values.

Some research from before the 1980s purposely included these wrong methodologies in their analyses. However, when applying mathematical operations to log-scaled signal values, they had a clear understanding of the definition and implications of using logarithmic power values. As they stated, they performed these methodologies to compare how different their results would be if using log-scaled signal values [34,35], or to explore the “*attractiveness of the logarithm of power*” such as its contribution to a compression of data requiring fewer bits of information [30] (what may have been a real concern at that time). Nevertheless, we did not find any discussion or argument on why to use log-scaled signal values in more recent papers. Indeed, many of these papers manipulated dBm values without mentioning the correspondence between dBm and watt values [13,15,20,21,27,36,37,38,39,40,41], and moreover, some of them manipulated signal-strength values without reporting the unit of measurement employed [12,14,29,42,43]. Many of the papers that followed these wrong methodologies got log-scaled signal-strength measurements directly from mobile operating systems (Android or iOS) [1,8,11,12,13,15,20,23,26,27,36,37,38,40]. Therefore, it is plausible that they just used and manipulated the data returned by the systems without a thorough analysis about the unit of the collected signal-strength values.

It is important to understand that applying mathematical operations with log-scaled signal-strength values involves wrong models and interpretations of reality, and therefore, leads to wrong conclusions. Nevertheless, related research works have frequently made these mistakes. Many of these papers have been published during the last few years, demonstrating that the misuse of log-scaled signal-strength values is a real problem within the mobile computing community presently. These methodologies must be avoided, even when they have been constantly used in the past, since their habitual use is not a valid argument against their contradiction with some basic principles of scientific analysis.

The following subsections describe some of the common, but misinterpreted practices.

### 3.1. Averaging Signal Strength

The average of signal-strength measurements taken in similar temporal space conditions has been widely employed. For instance, the arithmetic mean of measurements taken in a single point can be used to reduce measurement variance, since every signal-strength sample is assumed to be contaminated with unrelated additive noise. Moreover, the arithmetic mean of measurements inside the same geographic area can be used to obtain a representative value of signal strength, getting an estimation of the mathematical expectation of signal strength in the area (as shown in Section 4.1).

The arithmetic mean involves taking the sum of samples; however, we already stated the lack of physical sense and relevance of the addition (and any other equation) involving log-scaled signal values. Consequently, the arithmetic mean of log-scaled signal values cannot either be considered to be physically relevant. This bad practice implies in most cases a distortion of real signal-strength behavior [30,35], as shown in the following simple but explanatory example. Let *a* be a vector of signal-strength values in dBm units:

(4) $$a=\left[\begin{array}{cc} -45\;\mathrm{dBm} & -55\;\mathrm{dBm} \end{array}\right]$$

The arithmetic mean of samples in *a* (in linear scale) is 1.74e−5mW, which is equal to −47.6dBm. Instead, the arithmetic mean of log-scaled samples in *a* is −50dBm, with an error of 2.4dB from the real value introduced by this misleading methodology. Although these errors may seem small in some cases, they should not be underestimated due to the impact of signal-strength fluctuations on other important network performance metrics [1,2,3,4]. Differences around 5dB in signal strength could imply in some cases an increase of 100% in packet loss rate and round-trip time of a connection over the mobile network [3].

Despite the aforementioned, there are works in which several signal-strength samples were aggregated by performing a log averaging process, taking the arithmetic mean of dBm or ASU measurements, misunderstanding signal-strength real behavior. Some works that used this incorrect methodology are listed below:

- (2017) Sabu et al. [11] conducted a correlation study between signal strength and rainfall intensity in an area of interest, where logarithmic ASU values were aggregated by taking the arithmetic mean. As result, the authors concluded that the drop of signal strength during rainfall was not as significant as expected by the theoretical hypothesis.
- (2018) In the data exploration section provided by Sung et al. [8], an area of interest was divided in smaller square areas. For each square area, signal strength was reported by taking the mean of several logarithmic ASU measurements. As result, a weak geographical correlation between signal strength and throughput was found.
- (2015) In the research work of Marina et al. [13], signal-strength samples in dBm units were divided according to their context (indoor or outdoor), and then aggregated by taking the arithmetic mean. Then, the authors analyzed the great impact of user context (indoor or outdoor) on the received signal strength.
- (2013) Sonntag et al. [1] created signal-strength coverage maps by taking the arithmetic mean of values represented as percentages. The percentage values are calculated linearly from logarithmic signal strength, so they are also logarithmic values. The authors concluded that coverage maps created from crowdsourced signal strength were not very good at presenting the actual transport quality.

In addition to the above, there are other research papers that also followed the incorrect methodology of calculating the arithmetic mean of logarithmic signal-strength values [12,17,20,21,36,37,38,40].

The only recorded case in radio frequency analysis where directly average log-scaled measurements using the arithmetic mean is admitted, is when dealing with samples of a repetitive or continuous wave signal. For this case, averaging log-scaled values is equal to log-scaling the average of linear values [44]. However, this is not the case of the signals we are referring to in this paper. Therefore, the introduced error by averaging log-scaled values depends on the statistics of the power estimates being averaged [30,34]. Consequently, considering that log-scaling the linear average will be equal to the average of the log-scaled values, and that the introduced error can be ignored, are incorrect assumptions.

### 3.2. Comparing Signal Strength

When comparing two signal-strength values, e.g., to calculate a prediction error, it is essential to properly measure the difference between the values. For example, if the signal strengths we want to compare are −50dBm and −45dBm, then it is correct to say that the values differ in 5dB, which represents the relation between both signal strengths. It is also correct to say that the difference between these values is 2.162e−5mW (the difference in linear scale) or, equivalently, −46.65dBm, although this latter form in dBm units can be quite confusing. However, it is a big mistake to say that the difference between −50dBm and −45dBm is 5dBm, since this is equal to 3.162mW, which is several orders of magnitude greater than the real difference shown before. Despite the above, some works performed signal-strength comparisons using this last incorrect methodology [13,36,40]. This misunderstanding is demonstrated in sentences as “*median error is 6 dBm*” [40], “*differing by more than 15 dBm*” [13], “*the real signal strength is 2.5 dBm stronger*” [38] or “*the errors are 10 dBm, 7 dBm, and 6 dBm, respectively*” [36]. These error levels are not coherent with the data used, where there are practically no values greater than −50dBm.

In addition, when comparing different signal-strength prediction errors to decide which error is lower, it is very important to compare them using absolute errors (in watt or dBm scale) rather than relative errors (in dB). The comparison of prediction errors using relative values, could lead to misunderstandings illustrated in the following simple but explanatory example. An error of +4dB at predicting −70dBm could be considered lower than an error of +5dB at predicting −110dBm, holding that 4dB<5dB. However, if we analyze these errors as linear absolute errors, we find that the first error is actually more than 10,000 times greater than the second one, giving an absolutely opposite view than the obtained by analyzing relative dB values. An example of the use of this incorrect methodology is described in the following:

- (2019) Recently, Alimpertis et al. [45] proposed a new method based on machine learning to perform signal-strength prediction, i.e., given a set of signal-strength measurements in an area, estimate signal-strength values in other singular points. They claimed that their method consistently obtains lower prediction errors than related state-of-art algorithms. Nevertheless, it can be shown that the comparison of errors used by them leads to inconclusive results. Using the values shown in Table 4 of [45], for cell ID x204, their method obtains an average error of 2.3dB, outperforming Ordinary Kriging (OK) and Ordinary Kriging Detrending method (OKD) which obtains average errors of 3.85dB and 2.99dB respectively. However, if we consider the case in which their method’s error is +2.3dB, OK’s error is −3.85dB, and OKD’s error is −2.99dB, all in relation to the expectation of the signal strength of cellID x204 (−96dBm), we have that OK method has an error 26% lower than their method’s error, and OKD method has an error 30% lower than their method’s error (using linear watt scale). In that case (a possible case given the prediction errors stated in the paper), their method actually gets worse results than related state-of-art algorithms.

Another common task is to summarize several prediction errors for (real forecast) pairs of signal-strength values. This is performed by calculating different measures of prediction accuracy as MAE (Mean Absolute Error), MAPE (Mean Absolute Percentage Error), or MASE (Mean Absolute Scaled Error). Nevertheless, many of these works failed at estimating their prediction accuracy by using a logarithmic scale for prediction errors, and summarizing them by applying mean-based aggregation as MAPE [26,27], MSE (Mean Squared Error) [28], or RMSE (Root Mean Squared Error) [45]. Then, they added a source of error at applying mean functions to log-scaled values, as mentioned in Section 3.1. In fact, since it is well known that the average of the logs will always be less than or equal to the log of the average [35], applying mean-based error measures to log-scaled errors, will imply an underestimation of real errors.

Signal-strength samples in linear scale should be preferred for estimating the error between two signal-strength values and for summarize several errors in an accuracy measure. However, this does not prevent these results from being used latter in log-scale if desired (for example, for visualization).

## 4. Signal-Strength Aggregation

As mentioned in Section 1, we consider each aggregated value as an estimation of the mathematical expectation of signal strength:

For an area *A*, we consider the function $P(\vec{p})$, which represents the signal strength in function of the position $\vec{p}$. Thus, a representative signal-strength value for *A*, obtained from the aggregation of individual measurements, will try to be as close as possible to the mathematical expectation E(P(X)), where *X* is a uniformly distributed random variable of position in *A*.

We define ${\bar{P}}_{A}$ as the division of the integral of the function *P* in *A*, and the total area *A*, which is equal to E(P(X)) as shown below:

(5) $$E\left(P\left(X\right)\right)=\int_{\Omega }P\left(\omega \right)f_{X}\left(\omega \right)d\omega =\frac{{\;\int \int \;}_{A}P\left(\vec{p}\right)dA}{{\;\int \int \;}_{A}dA}=:{\bar{P}}_{A}$$

where Ω denotes the set of all positions ω in *A*, $f_{X}$ is the probability density function of *X*, which is a constant equal to $\frac{1}{{\;\int \int \;}_{A}dA}$, and $\vec{p}$ denotes the position in *A*.

Considering a discretization of the space, the mathematical expectation in Equation (5) can be approximated by Riemann sums:

(6) $${\bar{P}}_{A}\approx \frac{\sum_{i=1}^{m}P\left(x_{i}\right)\Delta A_{i}}{\sum_{i=1}^{m}\Delta A_{i}}$$

and, when the discretization is such that all the points are equispaced, it follows that $\Delta A_{i}=\Delta A$ is constant for all i=1,…,m. The approximation becomes better as ΔA gets smaller, and consequently, the number of points (denoted by $m_{\left(\Delta A\right)}$) gets larger. Accordingly, the approximation by Riemann sums corresponds to the arithmetic mean and fulfills that:

(7) $$\lim_{\Delta A\to 0}\frac{1}{m_{\left(\Delta A\right)}}\sum_{i=1}^{m_{\left(\Delta A\right)}}P\left(x_{i}\right)={\bar{P}}_{A}$$

Thus, as the equispaced discretization becomes finer, the better the approximation of ${\bar{P}}_{A}$. The main drawback for this approximation method is the need to know the value of *P* in several equispaced positions over *A* to obtain an accurate estimation.

Another strategy to approximate the expected value relies in considering that the positions of the measurements $x_{i},\;i=1,\ldots ,m$, are given by independent uniform random variables over the area *A*. Then, we can use the Monte Carlo method to approximate ${\bar{P}}_{A}$ and, by the law of large numbers, we have that

(8) $$\lim_{m\to \infty }\frac{1}{m}\sum_{i=1}^{m}P\left(x_{i}\right)={\bar{P}}_{A}$$

The expression in Equation (8) is equivalent to Equation (7), and corresponds to the arithmetic mean. This is the value that past works referred to as the “local mean signal strength”, used to summarize signal strength in areas of a few meters (up to 40 wavelengths) [16,17,18]. In fact, local mean signal strength “*is obtained by averaging a large number of individual RF measurements taken in a local neighborhood*” [16]. Thus, related studies that estimate local mean signal strength are actually estimating the mathematical expectation of signal strength.

In the following, we discuss algorithms for estimating ${\bar{P}}_{A}$, using data from Mobile Crowdsourcing apps. In the first place, we consider as aggregation methods two commonly used performance metrics: arithmetic mean and median value. In addition, we propose a novel method based on the interpolation of signal-strength values.

### 4.1. Arithmetic Mean

A simple method to summarize signal-strength measurements is to take the arithmetic mean ${\bar{x}}_{A}$ of all the samples in area *A*, as commonly used in Mobile Crowdsourcing contexts [1,8,11,13,16,29]:

(9) $${\bar{x}}_{A}=\frac{1}{n}\sum_{i=1}^{n}x_{i}$$

This is a good first approach to estimate ${\bar{P}}_{A}$, based on the fact that if the samples are independent uniformly distributed in area *A*, then ${\bar{x}}_{A}$ is an example of Monte Carlo method, which assures that ${\bar{x}}_{A}$ converges to ${\bar{P}}_{A}$ when n→∞, as shown in Equation (8). Indeed, the law of large numbers and the Monte Carlo method could apparently justify the use of the arithmetic mean as an estimator of the mathematical expectation of signal strength in area A. Nevertheless, research studies that used the arithmetic mean over signal-strength samples did not look over the fulfillment of the hypothesis required by the Monte Carlo method. First, in a realistic Mobile Crowdsourcing scenario, the number of signal-strength samples could be low for small areas. Second, crowdsourced signal-strength measurements would not be sampled uniformly on area A, as their positions are determined by human mobility patterns. Accordingly, there is no real mathematical foundation for using the arithmetic mean as estimator of the expected value of signal strength in these measurement contexts.

Estimating ${\bar{P}}_{A}$ by taking the arithmetic mean of Mobile Crowdsourcing data, is based on a *convenience sampling* process that only considers measurements from locations that are readily available or easy to reach. Readily available locations are directly defined by the mobility of test users. The use of this sampling method is well known to be likely to have biased results, because selecting cases based on their availability does not allow a generalization to the total population [46]. In our case, this means that the estimation of ${\bar{P}}_{A}$ will be biased by the locations in area *A* where test users took measurements.

As mentioned in Section 3.1, the arithmetic mean of signal samples should be taken over linear values to avoid induced bias due to incorrect methodologies. In addition to these physical and mathematical formalities, the importance of using linear values to better estimate the expected value of signal strength has also been stated in the past: “*In terms of accuracy, the preferred method for estimating the local mean signal strength at a specific point is to average (in **watts**) a **large number** of individual RF measurements*” [16]. Nevertheless, some works use the wrong methodology by explicitly applying the arithmetic mean over signal strength in logarithmic scale [1,8,11,13] as stated in Section 3.1.

Due to the above, in this paper we consider only the correct mean of samples in linear scale.

### 4.2. Median Value

Another method to summarize several signal-strength samples is by choosing the median value that separates the higher half from the lower half of all the measurements [10,47,48,49]. The idea behind using median value to aggregate signal-strength samples is that it is not skewed so much by a small proportion of extremely large or small values, which is a common situation in this case study, because, as shown in Section 3, signal amplitude could vary very widely among measurements.

Furthermore, since the logarithm is a strictly increasing function, the median value has the advantage that it will be the same value if selected from signal-strength values in linear (watt) or logarithmic (dBm) scale.

In cases as the Gaussian distribution, the median value is a good estimator for the mathematical expectation, since the latter naturally separates the higher from the lower half of possible values. Nevertheless, this assumption is not very likely to be true for Mobile Crowdsourcing contexts, where the signal-strength distribution depends on the positions of base transceiver stations with respect to the area of interest.

In addition to the above, the median value method also induces a bias due to the *convenience sampling* of measurements. Therefore, the median value is not expected to perform well at estimating the mathematical expectation of signal strength in Mobile Crowdsourcing scenarios, as there is no mathematical foundation for its use. However, due to its wide use in the literature, it is important to consider the median value as a baseline of our study to quantify the error it can reach at estimating ${\bar{P}}_{A}$.

### 4.3. Our Proposal: Average Based on Interpolation (ABOI Method)

As shown in Section 3.1, most Mobile Crowdsourcing scenarios do not fulfill the required hypotheses to employ the arithmetic mean as an estimator of the mathematical expectation of signal strength (hypotheses for Monte Carlo integration). Therefore, we wanted to design a more robust method to estimate the expected value from signal-strength measurements, without requiring the samples to be independent and uniformly distributed.

For our proposed method, we return to the idea of estimating the mathematical expectation of signal strength using Riemann sums, according to Equation (6). As mentioned in Section 1, to obtain better approximations to the real ${\bar{P}}_{A}$ by using Riemann sums, we need an equispaced grid of signal-strength values as fine-grained as possible. However, as stated in Section 2, it is not possible to ensure high number of measurements and uniform spatial distribution in most Mobile Crowdsourcing scenarios. To solve these problems, we use the available measurements to interpolate the signal strength in a fine-grained grid, obtaining equispaced data and increasing the number of available samples. Thus, to estimate ${\bar{P}}_{A}$ we take the arithmetic mean of all values in the fine-grained grid *G* (in watt) as shown in Equation (7), avoiding the difficulties of nonuniform spatial distribution and low number of measurements. Consequently, to obtain a good estimation of the mathematical expectation, we need to establish the conditions on the signal-strength measurements that guarantee a proper interpolation. As our proposed method is an average based on interpolation, we will refer to it as ABOI.

Although there are many interpolation methods, it is out of the scope of this paper discussing the advantages and disadvantages of each one. For the interpolation step in the ABOI method, we use one of the simplest and commonly employed interpolations methods in signal-strength analysis: the Ordinary Kriging (OK) algorithm. Nevertheless, the ABOI method could be improved by using a more complex and accurate interpolation algorithm.

To estimate the value of signal strength at a position $x_{0}$ on the grid, the OK algorithm takes a linear combination of its neighbors:

(10) $$P^{*}\left(x_{0}\right)=\sum_{i=1}^{n}\omega_{i}P\left(x_{i}\right)$$

where $x_{i}$ represents all the neighbors of $x_{0}$, and $\omega_{i}$ is the corresponding weight of each neighbor. In general, $\omega_{i}$ is proportional to the distance between $x_{0}$ and $x_{i}$.

It is important to notice that many authors wrongly used this method with dBm values or simply do not make explicit the scale used [36,41,43,45]. As mentioned in Section 3, we emphasize that this algorithm should be used on the linear power scale, since it involves algebraic operations, such as addition and weighting.

## 5. Mathematical Foundation for the Use of the ABOI Method

In this Section, we present the mathematical foundation for using the ABOI method to estimate the expected value ${\bar{P}}_{A}$ of signal-strength measurements inside an area A of interest. First, we announce Theorem 2, establishing the conditions under which the error of the estimation of ${\bar{P}}_{A}$ provided by the ABOI method can be smaller than ε. The hypotheses required for this result are shown to be consistent with realistic Mobile Crowdsourcing scenarios, contrarily to the case of arithmetic mean, as stated in Section 4.1. Lastly, we demonstrate that the ABOI method is an improvement on arithmetic mean at estimating ${\bar{P}}_{A}$, i.e., signal-strength measurements that are favorable for the arithmetic mean (that do fulfill Monte Carlo integration hypotheses) are still favorable for the ABOI method. However, favorable cases for the ABOI method can be very disadvantageous for the arithmetic mean.

### 5.1. ABOI Theorem

ABOI Theorem (Theorem 2) specifies the conditions under which the error of ABOI’s estimation can be smaller than ε, providing a proper approximation of the expected value of signal strength. For that purpose, some important definitions need to be stated first.

**Figure 1:**
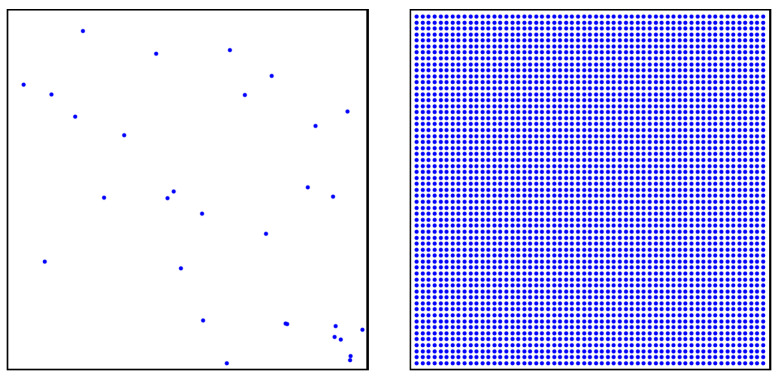
Example of set *N* with n=30 positions of initial measurements (**left**), and set *M* with m=3481 equispaced positions over *A* where to interpolate signal strength (**right**).

Let $N=\{x_{1},x_{2},\ldots ,x_{n}\}$ be the set of positions of the initial *n* signal-strength measurements taken inside a rectangle area $A=\left[a_{1},b_{1}\right]\times \left[a_{2},b_{2}\right]$. Analogously, let *M* be the set of positions of the *m* points equispaced over *A* on which the ABOI method interpolates signal strength. Sets *N* and *M* are exemplified in Figure 1.

Let us consider the following definition of the fill-distance:

(11) $$h_{N}:=\underset{x\in A}{\sup}\min_{x_{i}\in N}||x-x_{i}\left|\right|$$

The value $h_{N}$ indicates the largest distance between each position in *A* and its nearest neighbor in *N* (original measurements).

Let us call ABOI(*N*,*M*) the return value of the ABOI method after using the *n* original measurements to interpolate signal strength (using OK) on grid *M*, and computing the arithmetic mean of the *m* interpolated watt values.

**Theorem** **2.** *Given that power measurements P(·) can be modeled by Gaussian Processes (⋆). Let ε>0 be the desired error level for the estimation of the expected value provided by ABOI(N)(M). Let A be a rectangle area where to estimate the mathematical expectation of signal strength. If the n initial measurements are such that $h_{N}$ is small enough (⋆⋆), and selecting M as a fine-grained enough grid over A (⋆⋆⋆), then*

$$E\left[|E_{A}\left(P\left(X\right)\right)-\mathrm{ABOI}(N,M)|\right]\leq \varepsilon$$

*that is, the expected value of the error between the mathematical expectation of signal strength over A and the estimation provided by the ABOI method is smaller than the given ε.*

**Proof.**  Let $P_{M}$ be the arithmetic mean of real signal-strength values on each position in *M*. These *m* values are not known when applying the ABOI method. Nonetheless, $P_{M}$ will be helpful to bound the expected value of the estimation error. Indeed, we can bound the estimation error of the ABOI method as follows:

(12) $$\begin{array}{cc} \left|E_{A}\left(P\left(X\right)\right)-\mathrm{ABOI}(N,M)\right| & =|E_{A}\left(P\left(X\right)\right)-P_{M}+P_{M}-\mathrm{ABOI}(N,M)|\\ & \leq \underset{\left(I\right)}{\underset{︸}{\left|E_{A}\left(P\left(X\right)\right)-P_{M}\right|}}+\underset{\left(II\right)}{\underset{︸}{\left|P_{M}-\mathrm{ABOI}(N,M)\right|}} \end{array}$$

We will bound (I) and (II) separately.

$\left(I\right):\left|E_{A}\left(P\left(X\right)\right)-P_{M}\right|$

As stated in Equation (7), we can define $E_{A}\left(P\left(X\right)\right)$ as follows:

(13) $$E_{A}\left(P\left(X\right)\right)=\lim_{\Delta A\to 0}\frac{1}{m_{\left(\Delta A\right)}}\sum_{i=1}^{m_{\left(\Delta A\right)}}P\left(x_{i}\right)$$

or equivalently, for all ε>0, there exists a δ>0 such that

(14) $$\Delta A\leq \delta \Rightarrow |E_{A}\left(P\left(X\right)\right)-\frac{1}{m_{\left(\Delta A\right)}}\sum_{i=1}^{m_{\left(\Delta A\right)}}P\left(x_{i}\right)|<\varepsilon /2$$

where $\frac{1}{m_{\left(\Delta A\right)}}\sum_{i=1}^{m_{\left(\Delta A\right)}}P\left(x_{i}\right)$ is analogous to what we previously defined as $P_{M}$.

Therefore, hypothesis (⋆⋆⋆) allows us to select a fine-grained enough grid *M* that gives us the desired error bound ε/2.

Selecting *M* as aforementioned, we have that

(15) $$|E_{A}\left(P\left(X\right)\right)-P_{M}|\leq \varepsilon /2$$

$\left(II\right):\left|P_{M}-\mathrm{ABOI}(N,M)\right|$

As previously mentioned, $P_{M}$ is the arithmetic mean of real signal strength over *M* (*m* unknown values), whereas ABOI(N,M) is the arithmetic mean of the *m* interpolated values on grid *M* obtained by OK interpolation of the original *n* measurements in *N*. Therefore, $P_{M}$ and ABOI(N,M) are defined as follows:

$$\begin{array}{cc} P_{M} & =\left(\sum_{i=1}^{m}P\left(x_{i}\right)\right)/m\\ \mathrm{ABOI}(N,M) & =\left(\sum_{i=1}^{m}I_{N}\left(x_{i}\right)\right)/m \end{array}$$

where P(·) corresponds to the real signal strength, and $I_{N}\left(\cdot \right)$ corresponds to the OK interpolation of the original *n* signal-strength measurements in *N*. Accordingly,

$$\begin{array}{cc} \left|P_{M}-\mathrm{ABOI}(N,M)\right| & =|\left(\sum_{i=1}^{m}P\left(x_{i}\right)\right)/m-\left(\sum_{i=1}^{m}I_{N}\left(x_{i}\right)\right)/m|\\ & =|\left(\sum_{i=1}^{m}P\left(x_{i}\right)-I_{N}\left(x_{i}\right)\right)/m|\\ & \leq \left(\sum_{i=1}^{m}|P\left(x_{i}\right)-I_{N}\left(x_{i}\right)|\right)/m\\ & \leq \max_{i\in [1:m]}|P\left(x_{i}\right)-I_{N}\left(x_{i}\right)| \end{array}$$

Thus, the difference between $P_{M}$ and ABOI(N,M) is bounded by the maximum interpolation error among all the *m* positions of grid *M*. Wang et al. [50] provided an exhaustive analysis regarding this maximum interpolation error of OK. Indeed, hypothesis (⋆) allows the use of Corollary 1 of Wang et al. [50] along with Theorem 11.22 of Wendland [51] to obtain the following result (a detailed description of this outcome is provided in Appendix A):

(16) $$\lim_{h_{N}\to 0}E\left[\max_{i\in [1:m]}|P\left(x_{i}\right)-I_{N}\left(x_{i}\right)|\right]=0$$

or equivalently, for all ε>0, there exists a $\bar{h}$ such that

$$h_{N}\leq \bar{h}\Rightarrow E\left[\max_{i\in [1:m]}|P\left(x_{i}\right)-I_{N}\left(x_{i}\right)|\right]\leq \varepsilon /2$$

Therefore, hypothesis (⋆⋆) gives us the conditions such that $h_{N}$ is small enough to guarantee the desired error bound ε/2:

(17) $$E\left[|P_{M}-\mathrm{ABOI}(N,M)|\right]\leq \varepsilon /2$$

Finally, by joining the bounds for (I) and (II), i.e., by plugging (15) and (17) into (12), we obtain the desired inequality

$$E\left[|E_{A}\left(P\left(X\right)\right)-\mathrm{ABOI}(N,M)|\right]\leq \varepsilon$$

which completes the proof. □

### 5.2. Improvement on Arithmetic Mean

In this Section, we show that the ABOI method is an improvement on arithmetic mean at estimating ${\bar{P}}_{A}$. Both methods require specific conditions about the number and position of the initial signal-strength measurements. On the one hand, ABOI requires $h_{N}$ to be small enough (hypothesis (⋆⋆) of Theorem 2). On the other hand, the arithmetic mean requires the initial measurements to fulfill Monte Carlo integration hypotheses. Only if satisfying these conditions, the methods can be considered to be appropriate for estimating the mathematical expectation of signal strength. In the following, we will show that:

**Preposition** **2.1.** *If the initial measurements allow the arithmetic mean to be considered to be an appropriate estimator of ${\bar{P}}_{A}$, this implies that the ABOI method will also be considered to be an appropriate estimator of ${\bar{P}}_{A}$.*

**Preposition** **2.2.** *If the initial measurements allow the ABOI method to be considered to be an appropriate estimator of ${\bar{P}}_{A}$, this does not imply that the arithmetic mean will be considered to be an appropriate estimator of ${\bar{P}}_{A}$.*

**Proof** **of** **Preposition** **2.1.** If the initial conditions allow the arithmetic mean to be considered to be an appropriate estimator of ${\bar{P}}_{A}$, then the set of *n* signal-strength measurements fulfill Monte Carlo integration hypotheses (Section 4.1), i.e., the number *n* of measurements is high enough, and they are independent and uniformly distributed over the area. Theorem 6.6 of Niederreiter [52] suggests a bound for $h_{N}$ derived from its (extreme) discrepancy $D_{n}\left(N\right)$,

$$h_{N}\leq \sqrt{2}D_{n}^{1/2}\left(N\right)$$

where *N* is the set of positions of the *n* initial measurements. Given that the positions in *N* are independent random variables uniformly distributed over the area, Pronzato [53] states that

$$D_{n}\left(N\right)=O\left[{\left(\log n\right)}^{2}/n\right]$$

This result indicates that after a given number of measurements, hypothesis (⋆⋆) will be satisfied. Therefore, the ABOI method will also be considered to be an appropriate estimator of ${\bar{P}}_{A}$. □

The intuition behind Preposition 2.1 is that in case of measurements uniformly distributed over the area, both methods can be considered to be appropriate to estimate ${\bar{P}}_{A}$. However, as discussed in Section 1, uniform spatial distribution is an unrealistically optimistic case for crowdsourced measurements.

**Proof** **of** **Preposition** **2.2.** If the conditions allow the ABOI method to be considered to be an appropriate estimator of ${\bar{P}}_{A}$, then hypothesis (⋆⋆) is fulfilled. This hypothesis only requires $h_{N}$ to be small enough and does not require any specific distribution of measurements over the area. In particular, it does not require the measurements to be independent nor uniformly distributed over the area, which are necessary conditions for fulfilling Monte Carlo integration hypotheses. Therefore, the arithmetic mean may not be considered to be an appropriate estimator of ${\bar{P}}_{A}$. □

The intuition behind Preposition 2.2 is that the requirements of ABOI are less restrictive and more likely to be true in Mobile Crowdsourcing scenarios. As mentioned before, uniform spatial distribution is not a realistic case for measurements taken by real users, and therefore, there is no mathematical foundation for using the arithmetic mean (Section 4.1). However, real crowdsourced data is still able to fulfill hypothesis (⋆⋆), so far as the number of measurements allows it. Indeed, it is certainly expected that if the number of measurements is very low, then ABOI’s estimation will not be accurate, since $h_{N}$ will hardly be small enough. Likewise, if the number of signal-strength measurements is high, then ABOI’s estimation will be inclined to be closer to ${\bar{P}}_{A}$. This is a recurrent condition when estimating values of random effect models from measurements, and therefore, it cannot be avoided due to the stochastic behavior of the observations.

## 6. Experimental Results

Given that Section 5 provided the mathematical foundations for using the ABOI method to estimate the expected value of signal strength, we wanted to analyze experimentally its suitableness for this task. Additionally, we wanted to compare ABOI against the other aggregation methods commonly employed to estimate the mathematical expectation of signal strength (Section 4). In particular, we were interested in comparing ABOI with the arithmetic mean, as Section 5.2 gives us the intuition that the estimations provided by the ABOI method should be at least as good as the estimations provided by the arithmetic mean.

To evaluate and compare the aggregation methods described in Section 4, we performed experiments in both simulated and real scenarios. As this paper is the first attempt to challenge existing assumptions about signal-strength aggregation, we performed the following simplifications to the problem of estimating the mathematical expectation of signal strength in an area:

1. We considered areas with signal strength coming from only one base transceiver station (BTS).
2. Even when there may be a time variability of signal strength in the area [10,28], we considered that the mathematical expectation is estimated for a static power configuration of the BTS.

### 6.1. Simulated Scenario

We considered an area *A* of 500m×500m where a 30-meters tall BTS is placed at the center. We simulated the real signal strength on a fine-grained grid *G* over *A* with 5 m spacing, considering long-term attenuation due to path loss equation and medium-term variation due to shadowing modeled by a full covariance matrix [28,54,55,56,57]. Indeed, the real signal strength in *G* is given by

(18) $$\vec{1}P-10\alpha \log_{10}\left(\vec{d}\right)+\vec{v}$$

where $\vec{1}P={\left[P,P,\ldots ,P\right]}^{T}$ is a vector with *n* repeated values of *P*, the power transmitted by the simulated BTS; α corresponds to the path loss exponent; and $10\alpha \log_{10}\left(\vec{d}\right)$ is the path loss attenuation, where $\vec{d}={\left[d_{1},d_{2},\ldots ,d_{n}\right]}^{T}$ is the vector of distances between the position of each measurement and the position of the BTS. In addition, $\vec{v}$ is an attenuation factor due to shadowing effects, where

$$\vec{v}∼N\left(0,\Sigma_{v}\right)$$

and the covariance matrix $\Sigma_{v}$ is composed of elements given by $\mathrm{Cov}\left(x_{i},x_{j}\right)=\sigma_{v}^{2}\left(-d_{ij}/D_{corr}\right)$, where $d_{ij}$ is the distance between the positions $x_{i}$ and $x_{j}$ in *G*, and $D_{corr}$ is a parameter that models the correlation among the measurements.

Next, we simulated signal-strength measurements as if they were taken by real mobile devices, i.e., measurements included long-term attenuation due to path loss equation and medium-term variation due to shadowing, but they also included accuracy errors in both signal-strength values and geographic coordinates (due to hardware inaccuracy). The simulated measurements are given by

(19) $$\vec{X}=\vec{1}P-10\alpha \log_{10}\left(\vec{d}\right)+\vec{u}+\vec{v}+\vec{w}$$

where $\vec{X}={\left[x_{1},x_{2},\ldots ,x_{n}\right]}^{T}$ is an n×1 vector that contains the measurements. As in Equation (18), $\vec{v}$ is the attenuation factor due to shadowing effects. Additionally, as geolocation sensors are not perfectly accurate, position errors are considered when estimating the position of each measurement. This component is simulated by

$$\vec{u}∼N\left(0,\rho_{u}^{2}D\right),$$

which corresponds to a Gaussian distribution with a mean vector $\vec{0}$ and covariance matrix $\rho_{u}^{2}D$, where $D=\mathrm{diag}\{1/d_{1},1/d_{2},\ldots ,1/d_{n}\}$ [28]. Finally, $\vec{w}$ in Equation (19) is some unrelated additive noise, where

$$\vec{w}∼N\left(0,\sigma_{w}^{2}I_{n}\right).$$

For this simulation, the following values were used: P=−10 dBm, α=3.5, $\sigma_{w}=\sqrt{7}\;\mathrm{dB}$, $\sigma_{v}=\sqrt{10}\;\mathrm{dB}$, $\rho_{u}=0.2\;\mathrm{dB}$ and $D_{corr}=50\;m$. This setting is the same used by Santos et al. [28].

**Figure 2:**
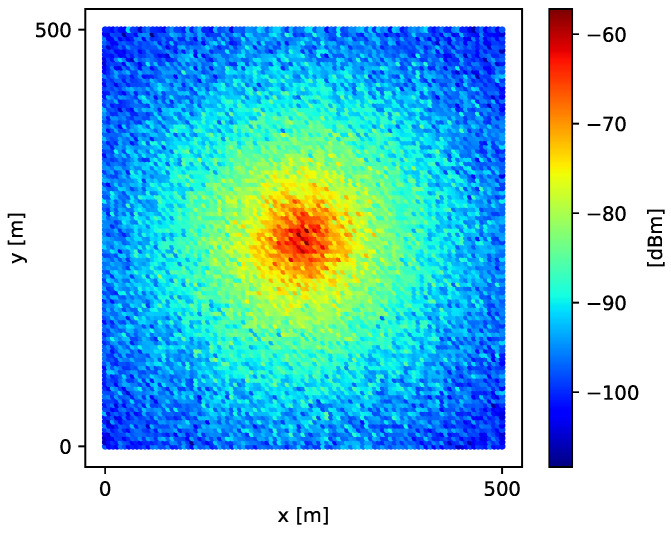
Simulated spatial field of signal strength over a fine-grained grid *G*.

Thus, signal-strength values simulated over grid *G* using Equation (19) generate the spatial field shown in Figure 2.

We calculated the ground truth ${\bar{P}}_{A}$ (expected value of signal strength over *A*) as a Riemann sum considering all values in *G*.

It is important to clarify that although the simulation model and its parameters were defined using dBm values, we always carefully manipulated signal-strength values using the linear watt scale. Thus, we avoided the mishandling of log-scaled signal-strength values, as discussed in Section 3.

For this experiment, we took different signal-strength measurement sets of sizes 50, 100, 200, 400, 700, and 1000. We distributed the samples on the grid by using two different methods:

1. Completely uniform distribution on the grid, which is commonly used, but not realistic for Mobile Crowdsourcing scenarios, as discussed in Section 1.
2. Considering the mobility model based on social network theory proposed by Musolesi et al. [19]. This model is closer to the spatial distribution of Mobile Crowdsourcing measurements, as they are defined by human mobility.

**Figure 3:**
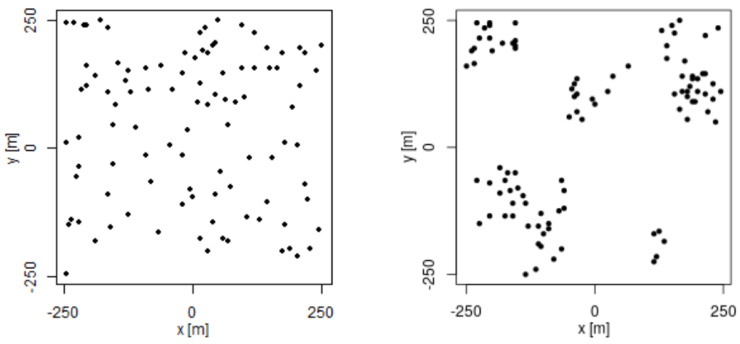
Example of spatial distribution for 100 samples using uniform distribution (**left**) and distribution based on social network theory (**right**).

Figure 3 shows the difference in spatial distribution of 100 samples at using the two methods explained above.

For every sample size and type of spatial distribution, we estimated ${\bar{P}}_{A}$ by applying the three methods presented in Section 4. We repeated each experiment 40 times, i.e., we took 40 different sample sets in every case.

**Figure 4:**
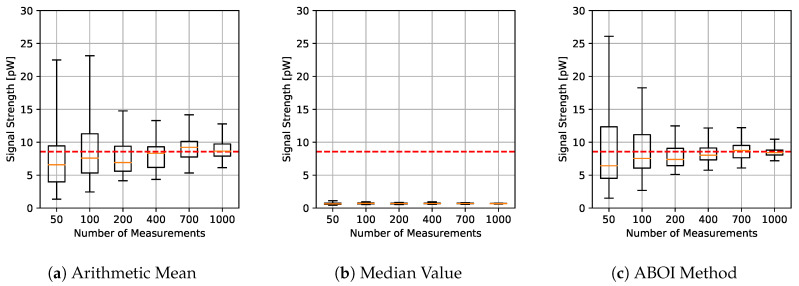
Simulated scenario. Boxplots for ${\bar{P}}_{A}$ prediction using the three aggregation methods and different sample sizes, selected by uniform distribution. Real ${\bar{P}}_{A}$ value in red line.

The results for experiments using uniform distribution are shown in Figure 4, where for each aggregation method and sample size we have the boxplot that depict the estimations of ${\bar{P}}_{A}$. It is important to clarify that all figures were calculated in linear scale, avoiding the errors mentioned in Section 3. All signal-strength values are shown in pW units, where 1 pW = $1\times 10^{-12}$ W.

As expected, arithmetic mean estimations tended to be close to ${\bar{P}}_{A}$, since uniform distribution is its best case, as explained in Section 4.1. Median value performed poorly, predicting nearly constant values far from the real one. Our proposed ABOI method showed satisfactory results and a similar behavior to the arithmetic mean.

**Figure 5:**
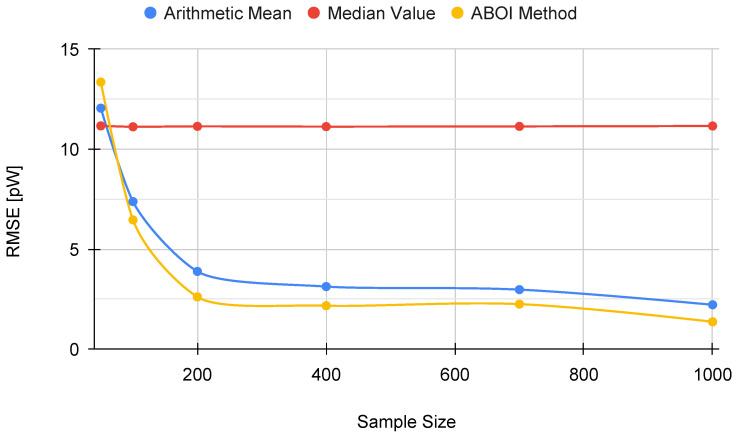
Simulated scenario. RMSE for ${\bar{P}}_{A}$ prediction for different sample sizes with uniform distribution.

In addition, Figure 5 shows the RMSE measure obtained by the aggregation methods, properly calculated using the linear values of the estimations of ${\bar{P}}_{A}$, as discussed in Section 3.2. RMSE values for our proposed ABOI rapidly decreased to low values, obtaining very similar results than the arithmetic mean.

These results agree with the intuition of Preposition 2.1, as in the case of uniform spatial distribution, both ABOI and the arithmetic mean performed well at estimating ${\bar{P}}_{A}$.

**Figure 6:**
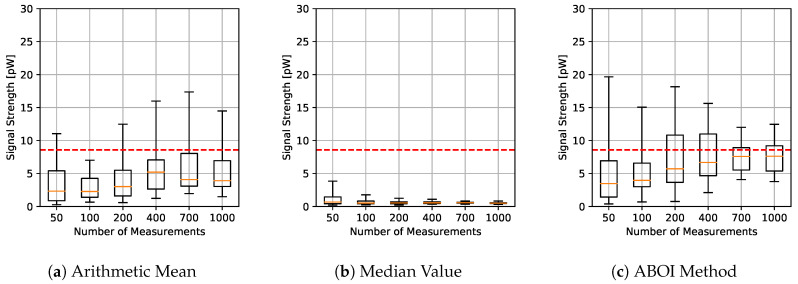
Simulated scenario. Boxplots for ${\bar{P}}_{A}$ prediction using the three aggregation methods and different sample sizes, selected by distribution based on social network theory. Real ${\bar{P}}_{A}$ value in red line.

The results for experiments using spatial distribution based on social network theory are shown in Figure 6. The arithmetic mean showed a more erratic performance than before, without a clear convergence to real ${\bar{P}}_{A}$ as the sample size increases. The median value showed similar behavior to the uniform distribution case, predicting nearly constant values. The ABOI method showed again a tendency to be close to real ${\bar{P}}_{A}$, but with a higher variability than for uniform distribution.

**Figure 7:**
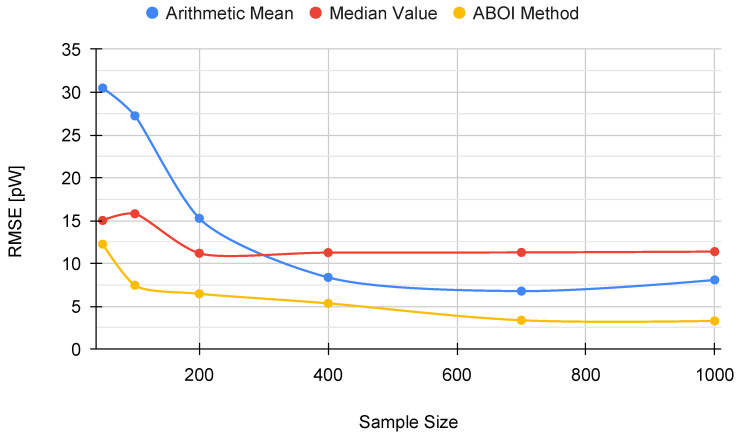
Simulated scenario. RMSE for ${\bar{P}}_{A}$ prediction for different sample sizes with distribution based on social network theory.

Figure 7 shows that our proposed ABOI method obtained consistently lower RMSE values than the other methods, with a remarkable improvement over arithmetic mean. Therefore, these experiments in a simulated scenario showed that the ABOI method is more reliable and more independent of the spatial distribution of samples at estimating the mathematical expectation of signal strength.

These results are also consistent with the mathematical foundations presented in Section 5, as ABOI performed well at estimating ${\bar{P}}_{A}$ in a nonuniform distribution scenario, which was close to the spatial distribution of crowdsourced measurements. In addition, as expected due to Proposition 2.2, spatial distribution based on social network theory did not satisfy the conditions required by the arithmetic mean to properly estimate ${\bar{P}}_{A}$.

### 6.2. Real Data

To test the aggregation methods using real data, we developed a very minimalist Android application to take signal-strength measurements with a densely time interval. The application was designed to run every 0.5 s. During each execution, the application used Android’s Telephony Manager to access information about the current cell being used by the device for network signaling. Thus, the Telephony Manager provided a CellIdentity object to obtain cell identifiers and a CellSignalStrength object to obtain the technology-specific signal strength in dBm. Along with this cell-related information, the application also stored the current location (latitude and longitude) with the highest accuracy possible.

**Figure 8:**
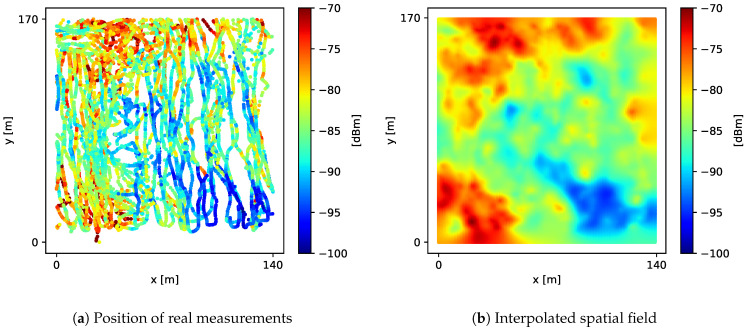
Real signal strength around the vicinity of a single LTE BTS. Color represents the dBm value of samples.

During a period of 2 consecutive hours, we took nearly 24,000 signal-strength measurements around the vicinity of a single LTE BTS (eNodeB) located in a residential area, using two different mobile devices. The received signal-strength measurements densely covered an area of 140m×170m near the BTS, as shown in Figure 8a. To calculate ${\bar{P}}_{A}$ value, we aligned the real measurements into a fine-grained grid *G* with 1 m spacing, obtaining the spatial field shown in Figure 8b. Then, we calculated the ground truth ${\bar{P}}_{A}$ as a Riemann sum of all values in *G*.

As with the simulation case, we performed experiments for different sample sets of sizes 25, 50, 100, 200, 350 and 500. We also considered both spatial distribution methodologies: uniform distribution and based on social network theory. As for the simulation experiments, we repeated each experiment 40 times.

**Figure 9:**
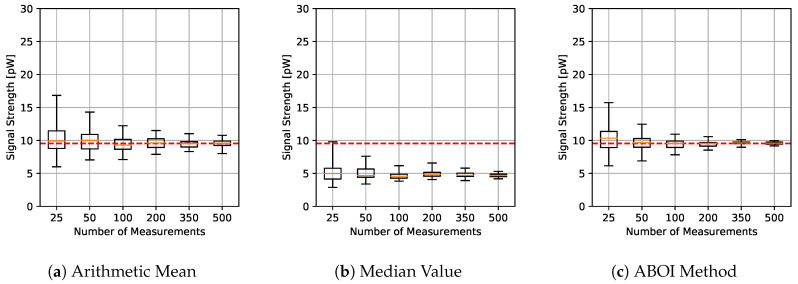
Real scenario. Boxplots for ${\bar{P}}_{A}$ prediction using the three aggregation methods and different sample sizes, selected by uniform distribution. Calculated ${\bar{P}}_{A}$ value in red line.

The results for experiments using uniform distribution are shown in Figure 9. We found that the behavior of the three methods was similar to the behavior shown by themselves in the simulation case with uniform distribution (Figure 4). The arithmetic mean and the ABOI method presented low and similar variability and a fast convergence to the calculated value of ${\bar{P}}_{A}$, where the ABOI method obtained slightly closer estimations to ${\bar{P}}_{A}$. The median value also showed coincident behavior with simulation case, predicting nearly constant and low values far from ${\bar{P}}_{A}$.

**Figure 10:**
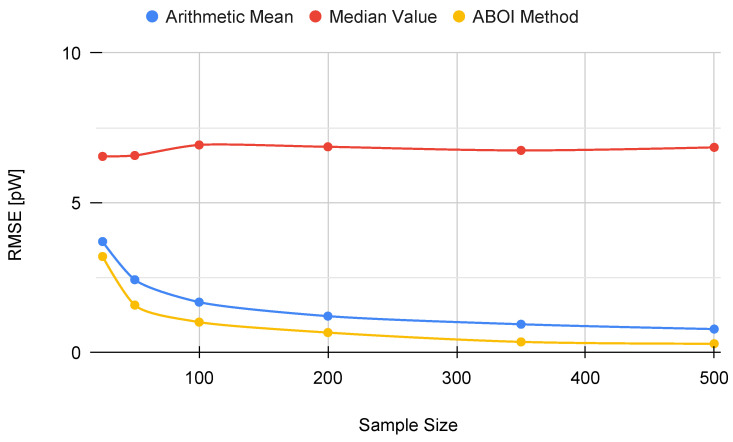
Real scenario. RMSE for ${\bar{P}}_{A}$ prediction for different sample sizes with uniform distribution.

Figure 10 confirms our analysis, as both arithmetic mean and our proposed ABOI method obtained similar RMSE values, outperforming the median value. It is important to remember that as stated in Section 4.1, uniform spatial distribution is the best case for the arithmetic mean, and therefore, its good performance was expected.

As for the simulated scenario, these results are coherent with the intuition of Preposition 2.1, as in the case of uniform spatial distribution, both ABOI and the arithmetic mean performed well at estimating ${\bar{P}}_{A}$.

**Figure 11:**
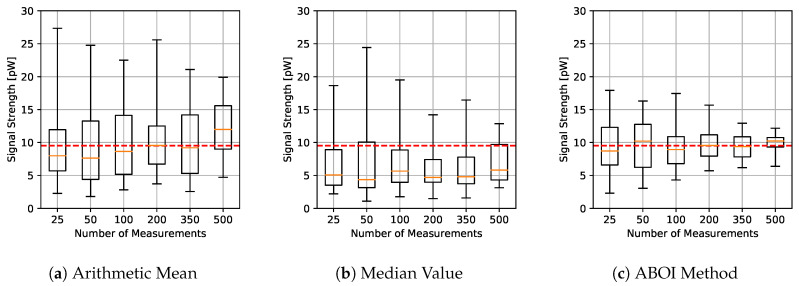
Real scenario. Boxplots for ${\bar{P}}_{A}$ prediction using the three aggregation methods and different sample sizes, selected by distribution based on social network theory. Calculated ${\bar{P}}_{A}$ value in red line.

The results for experiments with spatial distribution based on social network theory are shown in Figure 11. The arithmetic mean showed higher variability and worse estimations of ${\bar{P}}_{A}$ in relation to the previous case. The median value tended to predict low values. Our proposed ABOI method showed a similar behavior to the uniform distribution case, showing a clear convergence to ${\bar{P}}_{A}$. It also presented lower variability than the arithmetic mean.

**Figure 12:**
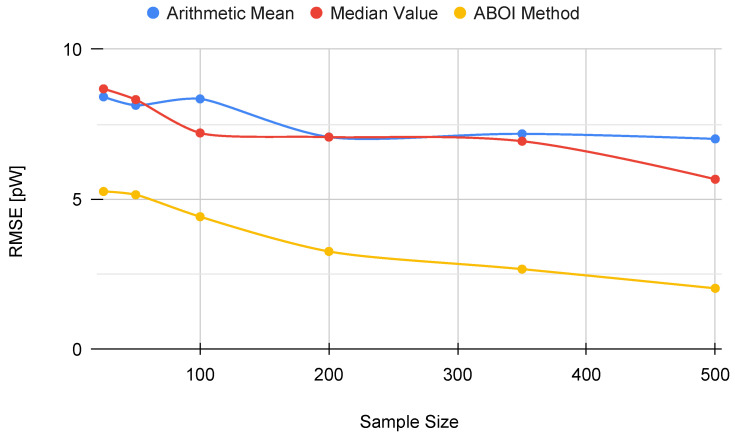
Real scenario. RMSE for ${\bar{P}}_{A}$ prediction for different sample sizes with distribution based on social network theory.

Figure 12 shows that the ABOI method obtained consistently lower RMSE values than the other methods, with a clear improvement over arithmetic mean. Unlike the arithmetic mean, our proposed method obtained more stable RMSE values at using both spatial distribution scenarios.

These results also agree with the mathematical foundations presented in Section 5 and with the obtained results in the simulated scenario, i.e., the spatial distribution based on social network theory allowed ABOI to perform well at estimating ${\bar{P}}_{A}$. In contrast, this spatial distribution did not satisfy the conditions required by the arithmetic mean to properly estimate ${\bar{P}}_{A}$.

Therefore, these experiments in a real scenario showed that our proposed method is more reliable and more independent of the spatial distribution of samples at estimating the mathematical expectation of signal strength.

Regarding the algorithms’ runtime performance, we measured their execution time using a 3.4 GHz quad-core processor (Intel${}^{®}$ Core${}^{\mathrm{TM}}$ i5-3570) with 12 GB RAM. For all methods, the execution time increased along with the number of measurements. On the one hand, the execution of the median value and the arithmetic mean never exceeded 0.01 s (both in the simulated case and in the real case). On the other hand, the execution of the ABOI method never exceeded 2 s. These results are an example of a well-known trade-off between estimation goodness and complexity. However, in our particular case, reducing the error at estimating ${\bar{P}}_{A}$ is much more relevant than reducing the time needed to compute the estimation, considering that the ABOI method’s runtime is still very low. Therefore, we do not consider the execution time as a drawback of our method.

File S1 contains all data and materials necessary for the reproducibility of experimental results. In addition, File S2 includes all the RMSE figures, but taking into account higher numbers of measurements, to visually clarify that the ABOI method will further reduce the error in our experiments.

## 7. Conclusions

In this paper, we first presented the physical and mathematical formalities about how signal-strength values must be handled at applying mathematical operations in a scientific and academic environment to avoid some common sources of error. We formally showed why some simple tasks as averaging and comparing signal-strength values are usually performed in contradiction with some scientific principles due to indiscriminate use of log-scaled values, which leads to errors in the analysis of experimental data, and therefore, to making wrong conclusions.

In addition, we presented a novel method based on interpolation to aggregate signal-strength samples into one representative value to estimate the mathematical expectation of signal strength in an area. This method is shown to present solid mathematical foundations to be employed on real Mobile Crowdsourcing scenarios.

Our proposed ABOI method outperformed other commonly used aggregation methods as arithmetic mean and median value, mainly because it was shown to be more independent of some Mobile Crowdsourcing data difficulties such as nonuniform spatial distribution of the samples, the potentially low number of measurements and the inaccuracy of end-user devices. By using this method, we computed more reliable estimations of the mathematical expectation of signal strength, in both simulated and real scenarios.

We conclude that for most Mobile Crowdsourcing scenarios, our proposed ABOI method should be preferred over the other methodologies.

As this paper was our first attempt to challenge existing assumptions about signal-strength aggregation, and to propose a novel method that performs better than other used algorithms, we performed some simplifications to the problem of estimating the mathematical expectation of signal strength in our experimental scenarios. However, we did consider some important challenges found in the wild, as long-term attenuation due to path loss, medium-term variation caused by obstacles in the area, and inaccuracy of end-users hardware, reflected on GPS locations and measured signal-strength levels. All these Mobile Crowdsourcing data difficulties were present in both simulated and real experimental scenarios.

As future work several related studies that followed some of the wrong methodologies presented in this paper can be repeated by properly handling signal-strength values. Therefore, we could investigate and quantify the induced impact on the results of an incorrect mathematical treatment. In addition, the ABOI method could be improved by considering more complex simulated scenarios, as areas with multiple antennas, and taking into account small scale fading caused by multipath propagation, and short-term attenuation fluctuations due to time variance in the channel. Additionally, as mentioned in Section 4.3, our proposed method could be improved by using a more complex and accurate interpolation algorithm.

## Data Availability

The data presented in this study are available in the supplementary material.

## Supplementary Materials

The following are available online at https://www.mdpi.com/1424-8220/21/4/1084/s1, File S1: Materials for reproducibility of experimental results, File S2: RMSE figures with higher number of measurements.

## Abbreviations

The following abbreviations are used in this manuscript:

QoS	Quality of Service
ABOI	Average Based on Interpolation
TCP	Transmission Control Protocol
BTS	Base Transceiver Station
ASU	Arbitrary Strength Unit
OK	Ordinary Kriging
OKD	Ordinary Kriging Detrending
MAE	Mean Absolute Error
MAPE	Mean Absolute Percentage Error
MASE	Mean Absolute Scaled Error
MSE	Mean Square Error
RMSE	Root Mean Squared Error
LTE	Long-Term Evolution

## Appendix A. Proof of Mathematical Expression (16)

It is clear that

(A1) $$E\left[\max_{i\in [1:m]}|P\left(x_{i}\right)-I_{N}\left(x_{i}\right)|\right]\leq E\left[\underset{x\in A}{\sup}|P\left(x\right)-I_{N}\left(x\right)|\right]$$

Then, Corollary 1 of Wang et al. [50] holds that selecting p=1,

$$E\left[\underset{x\in A}{\sup}|P\left(x\right)-I_{N}\left(x\right)|\right]=O\left(P_{\Phi ,X}\log^{1/2}\left(1/P_{\Phi ,X}\right)\right),\;\mathrm{as}\;P_{\Phi ,X}\to 0$$

It can be noticed that function $f\left(x\right)=x\log^{1/2}\left(1/x\right)$ satisfies the following limit:

$$\lim_{x\to 0^{+}}f\left(x\right)=0$$

Therefore,

(A2) $$\lim_{P_{\Phi ,X}\to 0^{+}}E\left[\underset{x\in A}{\sup}|P\left(x\right)-I_{N}\left(x\right)|\right]=0$$

Considering that power measurements P(·) over a rectangle area *A* can be modeled by Gaussian Processes (hypothesis (⋆) of Theorem 2), then Theorem 11.22 of Wendland [51] states that *exist positive constants c and $h_{0}$ depending only on A such that*$P_{\Phi ,X}\leq h_{N}^{c/h_{N}}$*provided that* $h_{N}\leq h_{0}$.

It can be seen that function $f\left(x\right)=x^{c/x}$ satisfies the following limit:

$$\lim_{x\to 0^{+}}f\left(x\right)=0$$

Consequently, it can be verified that

$$\lim_{h_{N}\to 0^{+}}h_{N}^{c/h_{N}}=0$$

and taking into account that Theorem 11.22 of Wendland [51] states that $P_{\Phi ,X}\leq h_{N}^{c/h_{N}}$, we have

$$h_{N}\to 0^{+}\Rightarrow P_{\Phi ,X}\to 0^{+}$$

Then, Equation (A2) can be rewritten in terms of $h_{N}$ as

(A3) $$\lim_{h_{N}\to 0^{+}}E\left[\underset{x\in A}{\sup}|P\left(x\right)-I_{N}\left(x\right)|\right]=0$$

Finally, by plugging (A3) into (A1) we obtain the desired expression in Equation (16):

$$\lim_{h_{N}\to 0}E\left[\max_{i\in [1:m]}|P\left(x_{i}\right)-I_{N}\left(x_{i}\right)|\right]=0$$
