# Supplementary material for: Improving Signal-Strength Aggregation for Mobile Crowdsourcing Scenarios
Source: Sensors (Basel). 2021 Feb 5;21(4):1084. doi: 10.3390/s21041084 (PMC7914496; doi:10.3390/s21041084)
Supplement: Supplementary file 1 [file sensors-21-01084-s001.zip › File S2 RMSE figures with higher number of measurements.pdf]

RMSE Figures With Higher Number of Measurements

Improving Signal Strength Aggregation for Mobile Crowdsourcing Scenarios

Diego Madariaga, Javier Madariaga,
Javier Bustos-Jiménez, and Benjamin Bustos

NIC Chile Research Labs, University of Chile
Contact: diego@niclabs.cl

6 Experimental Results

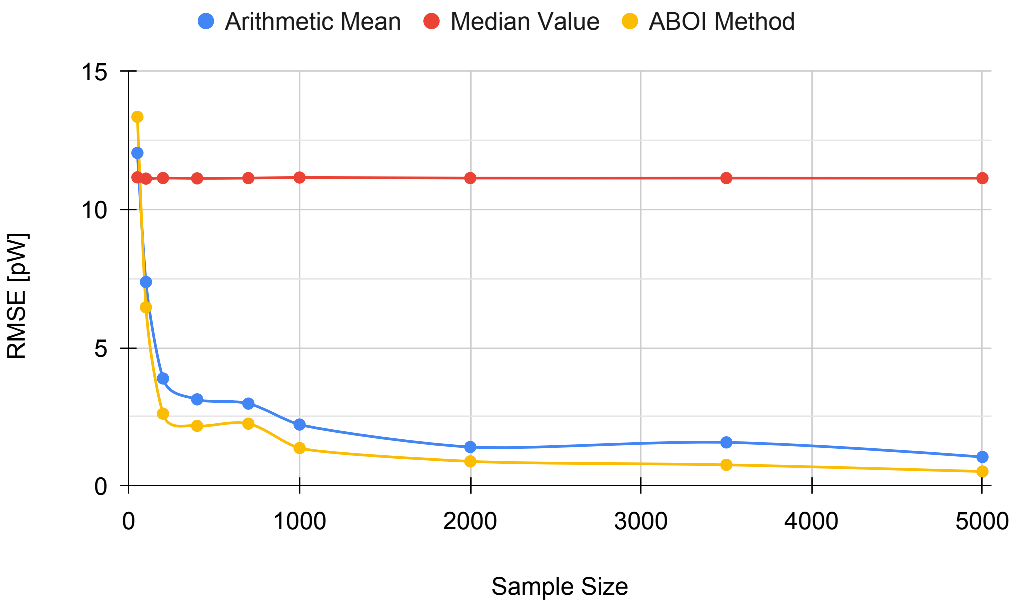

Fig. 5. Simulated scenario. RMSE for $\overline{P}_A$ prediction for different sample sizes with uniform distribution.

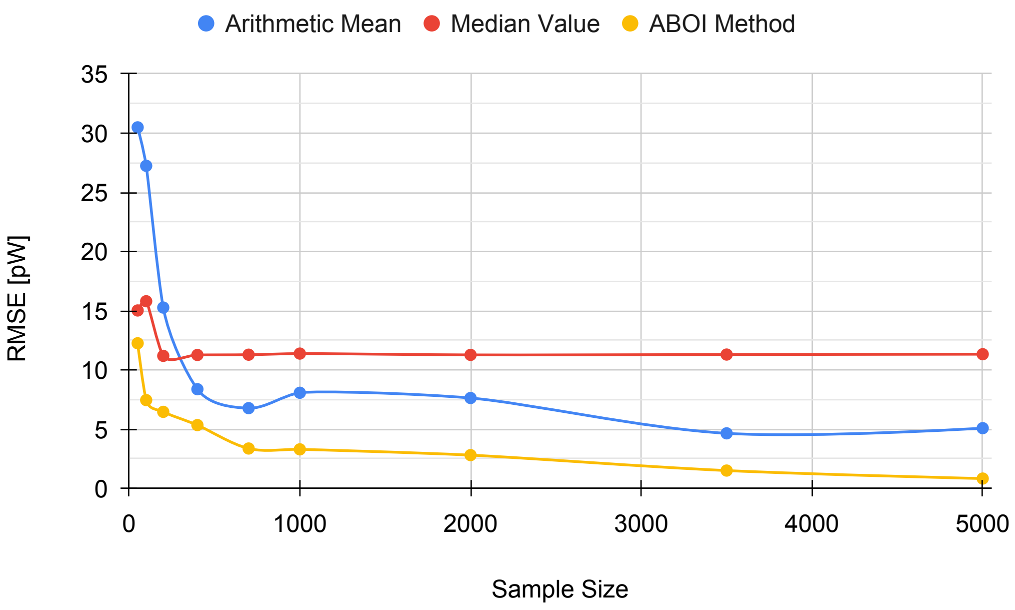

Fig. 7. Simulated scenario. RMSE for $\overline{P}_A$ prediction for different sample sizes with distribution based on social network theory.

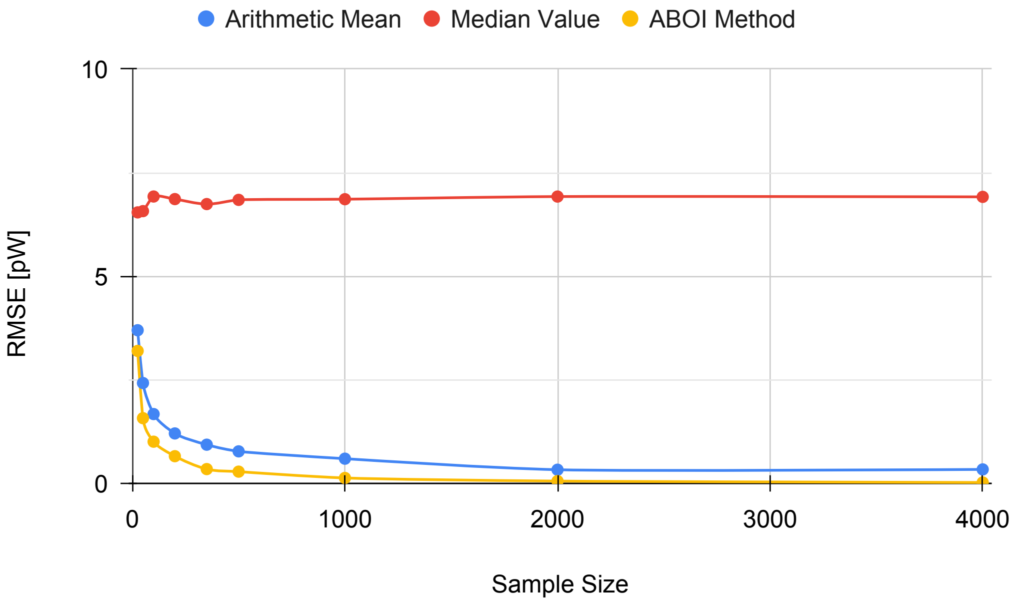

Fig. 10. Real scenario. RMSE for $\overline{P}_A$ prediction for different sample sizes with uniform distribution.

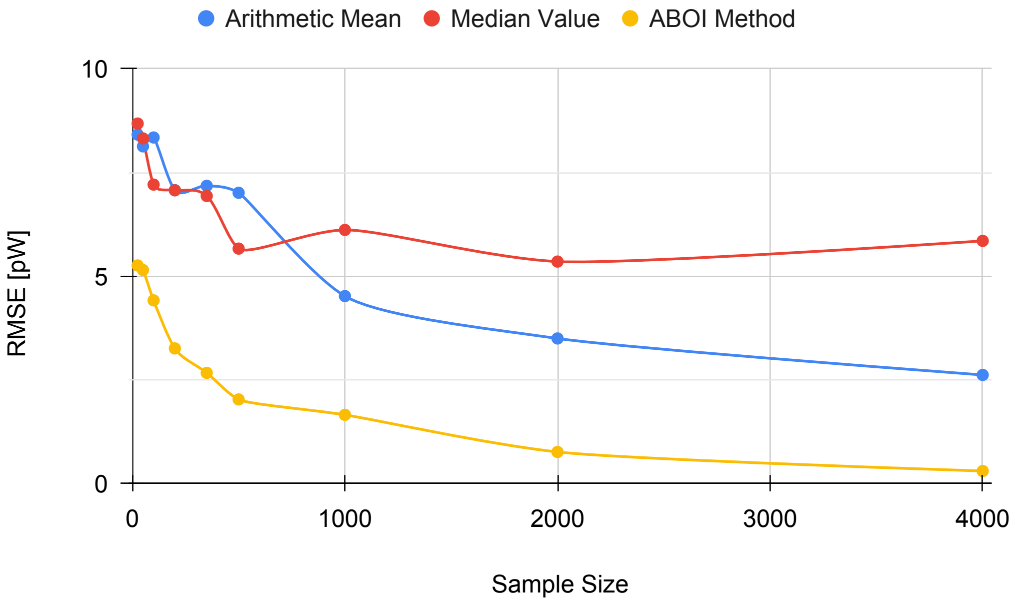

Fig. 12. Real scenario. RMSE for $\overline{P}_A$ prediction for different sample sizes with distribution based on social network theory.
