# Supplementary material for: Improving Signal-Strength Aggregation for Mobile Crowdsourcing Scenarios
Source: Sensors (Basel). 2021 Feb 5;21(4):1084. doi: 10.3390/s21041084 (PMC7914496; doi:10.3390/s21041084)
Supplement: Supplementary file 1 [file sensors-21-01084-s001.zip › File S1 Materials for reproducibility of experimental results.zip!/Materials_for_reproducibility_of_experimental_results/reproducibility_of_experimental_results.pdf]

Reproducibility of Experimental Results

Improving Signal Strength Aggregation for Mobile Crowdsourcing Scenarios

Diego Madariaga, Javier Madariaga,
Javier Bustos-Jiménez, and Benjamin Bustos

NIC Chile Research Labs, University of Chile
Contact: `diego@niclabs.cl`

1 Experiments for Simulated Scenario

The CSV file `simulated_ground_truth.csv` contains the ground truth spatial field of signal strength for the simulated scenario. Each of its rows contains a 3-tuple $(x\ y\ dbm)$, where $x \in \{0, ..., 500\}$, $y \in \{0, ..., 500\}$ and dbm corresponds to the signal strength value (in dBm) at position (x, y).

The folder `simulated_uniform/` contains the CSV files that describe the positions of measurements over `simulated_ground_truth.csv` distributed uniformly. The folder contains 240 files named `positions_X_Y.csv`, where $\texttt{X} \in \{50, 100, 200, 400, 700, 1000\}$ is the number of positions in the file and $\texttt{Y} \in \{0, ..., 39\}$ identifies each repetition of the experiment. These files can be generated by executing the following Python script:

```
$ cd simulated_uniform/
$ python simulated_positions_uniform.py
```

The Python scripts `simulated_positions_uniform.py` makes use of NumPy library.

Similarly, the folder `simulated_nonuniform/` contains the CSV files that describe the positions of measurements over `simulated_ground_truth.csv` selected by distribution based on social network theory. The folder contains 240 files named `positions_X_Y.csv`, where $\texttt{X} \in \{50, 100, 200, 400, 700, 1000\}$ is the number of positions in the file and $\texttt{Y} \in \{0, ..., 39\}$ identifies each repetition of the experiment. These files can be generated by executing the following R script:

```
$ cd simulated_nonuniform/
$ Rscript simulated_positions_nonuniform.R
```

The R script `simulated_positions_nonuniform.R` makes use of the `movGen` script implemented by Musolesi et al. [1], and therefore, it needs to be downloaded beforehand.

The following command generates Figure 4 of the paper[1]:

```
$ python experiments.py simulated uniform
```

The previous script creates 3 PDF files with the images for Figure 4. In addition, it prints to standard output all RMSE values needed for creating Figure 5.

The following command generates Figure 6 of the paper:

```
$ python experiments.py simulated nonuniform
```

The previous script creates 3 PDF files with the images for Figure 6. In addition, it prints to standard output all RMSE values needed for creating Figure 7.

[1] Python script `experiments.py` makes uses of the following libraries: NumPy, Pandas, PyKrige and Matplotlib.

2 Experiments for Real Scenario

The CSV file `real_ground_truth.csv` contains the ground truth spatial field of signal strength for real scenario. Each of its rows contains a 3-tuple $(x\ y\ dbm)$, where $x \in \{0, ..., 139\}$, $y \in \{0, ..., 169\}$ and dbm corresponds to the signal strength value (in dBm) at position (x, y).

The folder `real_uniform/` contains the CSV files that describe the positions of measurements over `real_ground_truth.csv` distributed uniformly. The folder contains 240 files named `positions_X_Y.csv`, where $\texttt{X} \in \{25, 50, 100, 200, 350, 500\}$ is the number of positions in the file and $\texttt{Y} \in \{0, ..., 39\}$ identifies each repetition of the experiment. These files can be generated by executing the following Python script:

```
$ cd real_uniform/
$ python real_positions_uniform.py
```

The Python scripts `real_positions_uniform.py` makes use of NumPy library.

Similarly, the folder `real_nonuniform/` contains the CSV files that describe the positions of measurements over `real_ground_truth.csv` selected by distribution based on social network theory. The folder contains 240 files named `positions_X_Y.csv`, where $\texttt{X} \in \{25, 50, 100, 200, 350, 500\}$ is the number of positions in the file and $\texttt{Y} \in \{0, ..., 39\}$ identifies each repetition of the experiment. These files can be generated by executing the following R script:

```
$ cd real_nonuniform/
$ Rscript real_positions_nonuniform.R
```

The R script `real_positions_nonuniform.R` makes use of the `movGen` script implemented by Musolesi et al. [1], and therefore, it needs to be downloaded beforehand.

The following command generates Figure 10 of the paper:

```
$ python experiments.py real uniform
```

The previous script creates 3 PDF files with the images for Figure 10. In addition, it prints to standard output all RMSE values needed for creating Figure 11.

The following command generates Figure 12 of the paper:

```
$ python experiments.py real nonuniform
```

The previous script creates 3 PDF files with the images for Figure 12. In addition, it prints to standard output all RMSE values needed for creating Figure 13.

References

1. Musolesi, M., Mascolo, C.: Designing mobility models based on social network theory. ACM SIGMOBILE Mobile Computing and Communications Review **11**(3), 59–70 (2007)
